# Purification and Partial Characterization of Trypsin-Specific Proteinase Inhibitors from Pigeonpea Wild Relative *Cajanus platycarpus* L. (*Fabaceae*) Active against Gut Proteases of Lepidopteran Pest *Helicoverpa armigera*

**DOI:** 10.3389/fphys.2016.00388

**Published:** 2016-09-07

**Authors:** Marri Swathi, Prashant K. Mishra, Vadthya Lokya, Vanka Swaroop, Nalini Mallikarjuna, Aparna Dutta-Gupta, Kollipara Padmasree

**Affiliations:** ^1^Department of Plant Sciences, School of Life Sciences, University of HyderabadHyderabad, India; ^2^Department of Biotechnology and Bioinformatics, School of Life Sciences, University of HyderabadHyderabad, India; ^3^Legumes Cell biology, Grain Legumes Program, ICRISATHyderabad, India; ^4^Department of Animal Biology, School of Life Sciences, University of HyderabadHyderabad, India

**Keywords:** gelatin activity staining, Kunitz trypsin inhibitor, mass spectrometry, miraculin-like proteins, two-dimensional electrophoresis

## Abstract

Proteinase inhibitors (PIs) are natural defense proteins of plants found to be active against gut proteases of various insects. A pigeonpea wild relative *Cajanus platycarpus* was identified as a source of resistance against *Helicoverpa armigera*, a most devastating pest of several crops including pigeonpea. In the light of earlier studies, trypsin-specific PIs (CpPI 63) were purified from mature dry seeds of *C. platycarpus* (ICPW-63) and characterized their biochemical properties in contributing to *H. armigera* resistance. CpPI 63 possessed significant *H. armigera* gut trypsin-like proteinase inhibitor (HGPI) activity than trypsin inhibitor (TI) activity. Analysis of CpPI 63 using two-dimensional (2-D) electrophoresis and matrix assisted laser desorption ionization time-of-flight (MALDI-TOF) mass spectrometry revealed that it contained several isoinhibitors and small oligomers with masses ranging between 6 and 58 kDa. The gelatin activity staining studies suggest that these isoinhibitors and oligomers possessed strong inhibitory activity against *H. armigera* gut trypsin-like proteases (HGPs). The N-terminal sequence of the isoinhibitors (pI 6.6 and pI 5.6) of CpPI 63 exhibited 80% homology with several Kunitz trypsin inhibitors (KTIs) as well as miraculin-like proteins (MLPs). Further, modification of lysine residue(s) lead to 80% loss in both TI and HGPI activities of CpPI 63. In contrast, the TI and HGPI activities of CpPI 63 were stable over a wide range of temperature and pH conditions. The reported results provide a biochemical basis for pod borer resistance in *C. platycarpus*.

## Introduction

Pigeonpea (*Cajanus cajan*) is an important pulse crop which occupies a major share in contributing to protein rich diet, particularly in the vegetarian population. India is the largest producer and consumer of pigeonpea in the world. Pigeonpea seeds contain extensive defense machinery which includes lectins, proteinase inhibitors (PIs) of trypsin and chymotrypsin, amylases, and secondary metabolites (Giri and Kachole, 1998; Prasad et al., 2009, 2010; Brito et al., 2012). In spite of the existence of such a broad spectrum of defense compounds, pre-harvest devastation due to insect pests in developing seeds and post-harvest losses caused by storage pests in mature seeds are severe in pigeonpea (Giri and Kachole, 1998; Sharma, 2016). Approximately, more than 200 insect pests are known to cause such type of damage to pigeonpea. *Helicoverpa armigera* is one among the most devastating insect pests on pigeonpea. The larvae of *H. armigera* grow on reproductive structures of pigeonpea and cause an estimated loss of US$2 billion in the grain yield of semi-arid tropics (Sharma, 2005). Also, most of the pesticide use worldwide is directed against *H. armigera* (Ahmad, 2007). However, several characteristic features of *H. armigera* such as wide geographical distribution, high polyphagy and fecundity, and propensity to develop resistance to insecticides contributed strongly for its adaptation to various cropping systems (Rajapakse and Walter, 2007). Thus, it has become a major challenge to control *H. armigera*. In this context, agronomic practices and natural enemies along with host plant resistance and natural plant products which are relatively harmless to the non-target beneficial organisms as well as human beings were considered to offer a potentially reasonable means of controlling insect pests (Andow, 2008).

PIs are ubiquitously distributed in plants (Padul et al., 2012), animals (Espana et al., 2007), and microbes (Sabotiè and Kos, 2012). They are classified mainly as serine, cysteine, aspartic and metallo PIs. The Kunitz and Bowman-Birk inhibitors (BBIs) of serine PIs are well characterized and gained importance as biopesticides. The Kunitz-type inhibitors are ~19 kDa proteins with two disulfide linkages whereas BBIs are usually ~8 kDa proteins with seven disulfide bridges (Macedo et al., 2015). The PIs are known to influence the growth and development of insects by binding tightly and irreversibly to the active site of its digestive gut proteases, which are essential for various metabolic processes such as protein turnover or proteolytic digestion (Richardson, 1991). This mechanism leads to a critical amino acid deficiency and eventually insect death due to over production of digestive proteases by diverting the essential amino acids available for the production of other proteins (Zhu-Salzman and Zeng, 2015).

The PIs present in storage tissues and leaves of plants are induced upon insect wounding, thereby significantly reducing the attack of insect pests on plants (Ryan and Moura, 2002). Thus, they are produced as plant's own natural defense proteins against phytophagous insects. Also, the use of PIs in generating insect resistant transgenic plants is beneficial as they protect other natural defense proteins of plants from proteolytic degradation during insect invasion (Sharma, 2015). Thus, introduction of PI genes as compared to other defense genes in transgenic plants would prevent economic losses worldwide due to insect pests (Dunse et al., 2010; Chen et al., 2014; Quilis et al., 2014). However, insects show several adaptive mechanisms to overcome from PI mediated defense exhibited by plants: (i) over-expression of expressing gut proteases to compensate for the loss in activity of digestive proteases (De Leo et al., 1998; Ahn et al., 2004); (ii) synthesis of new gut proteases which are resistant against ingested PIs (Oppert et al., 2005) and (iii) activation of gut proteases that hydrolyze plant PIs (Yang et al., 2009). In this context of host-pest co-evolution, identification of new plant PIs, particularly from wild relatives with prominent inhibitory potential against gut proteases of *H. armigera* have to be continuously screened, identified and examined for their biochemical properties to successfully utilize them as biopesticides by external application or through transgenic technology (Mulimani and Sudheendra, 2002; Parde et al., 2012).

Wild relatives of pigeonpea are known to possess PIs active against insect pests such as *H. armigera, Spodoptera litura*, and *Achaea janata* (Chougule et al., 2003; Prasad et al., 2009; Parde et al., 2012; Swathi et al., 2014). *Cajanus platycarpus* belonged to *Fabaceae* family and is one among the tertiary gene pool of wild relatives. It has the same chromosome number as that of cultivated pigeonpea (*2n* = 22) which is now acquiescent to interspecific hybridization and gene transfer (Mallikarjuna et al., 2006). It has various traits of interest such as extra-early flowering and maturity, photoperiod insensitivity, prolific flowering and podding, high harvest index, annuality and rapid seedling growth. Also, it has been recognized as a source of defense genetic base not only against *H. armigera* (Saxena et al., 1996; Sujana et al., 2008), but also against phytophthera blight (Chauhan et al., 2002), nematodes (Sharma, 1995), and sterility mosaic virus (Kumar et al., 2005). Keeping in view the severe losses caused in pigeonpea by *H. armigera* worldwide, efforts have been made to introgress the potential novel defense genes from *C. platycarpus* into the genome of cultivated pigeonpea varieties with a narrow genetic base and susceptibility to pests (Mallikarjuna et al., 2006, 2011; Mallikarjuna, 2007).

The *in vitro* studies of Swathi et al. (2015) revealed the presence of PIs active against *H. armigera* gut trypsin-like proteases (HGPs) in various plant organs such as leaves, flowers, pods, developing, and mature seeds of *C. platycarpus* accessions (ICPW 60-72). ICPW-63 is one among the tested accessions which possessed HGPIs with strong inhibitory activity against HGPs. Since trypsin-like proteases predominated over chymotrypsin-like proteases in the midgut of *H. armigera*, in the present study, trypsin specific PIs (CpPI 63) are purified from *C. platycarpus* accession ICPW-63 by applying various chromatographic techniques. Further, the biochemical properties such as detection of isoinhibitors, self-association pattern of CpPI 63 and its stability against the proteolytic activity of HGPs was examined in a wide range of temperature and pH conditions. Furthermore, application of mass spectrometry studies and N-terminal sequencing paved path for the putative identification of two isoinhibitors of CpPI 63 separated by 2-D gel electrophoresis. Thus, this study complements the earlier findings of Swathi et al. (2015) in characterizing the *C. platycarpus* PIs active against *H. armigera* by various methods.

## Materials and methods

Bovine serum albumin (BSA) and bovine pancreatic trypsin were procured from Sisco Research Laboratory (Mumbai, India). DEAE-cellulose, trypsin-Sepharose 4B, Sephadex G-50, N-α-benzoyl-DL-arginine-*p*-nitroanilide (BA*p*NA), Bowman-Birk inhibitor from soybean (BBI), sorbitol, tricine, gelatin, and coomassie brilliant blue (CBB) R-250 were purchased from Sigma (St. Louis, MO). Immobilized pH gradient (IPG) strips (pH 4–7 linear, 11 cm), IPG buffer (pH 4–7 linear), dithiothreitol (DTT), and iodoacetamide (IDA) were procured from GE Healthcare Bio-Sciences AB (Uppsala, Sweden). Bicinchoninic acid (BCA) protein estimation kit was purchased from Thermo Scientific (USA). The protein molecular mass standards (range 3.5–57 and 4.6–100 kDa) from Puregene, Genetix, India were used. All other chemicals and reagents used were of analytical grade.

### Seed material and insects

Mature dry seeds of pigeonpea wild relative *C. platycarpus* accession, ICPW-63 were obtained from the International Crops Research Institute for Semi-Arid Tropics (ICRISAT), Hyderabad, India. *H. armigera* insects were obtained from the National Bureau of Agriculturally Important Insects (NBAII), Bangalore, India.

### Preparation of crude PI extract

The seed powder prepared from mature dry seeds was extracted into 50 mM Tris-HCl, pH 8.0 containing 1% polyvinylpyrrolidone in 1:6 (w/v) ratio under mild stirring continuously for overnight at 4°C as described in Prasad et al. (2009). The supernatant obtained after centrifuging twice at 12,880 g for 20 min (4°C) was used as a crude PI protein extract.

### Purification of trypsin specific proteinase inhibitors (CpPI 63)

The crude PI protein extract described above was subjected to 0–20, 20–70 and 70–100% (NH_4_)_2_SO_4_ fractionation for 1 h (4°C). The corresponding protein precipitates were solubilized, dialyzed against 3.0 kDa cut-off membrane filters (Millipore Corporation) using 50 mM Tris-HCl, pH 8.0 and protein content (Smith et al., 1985), and trypsin inhibitor (TI) activity were determined. The 20–70% (NH_4_)_2_SO_4_ fraction with maximum TI activity was purified by passing sequentially through the DEAE-cellulose column (2.2 × 20 cm), trypsin-Sepharose 4B column (1.3 × 15 cm), and Sephadex G-50 column (1.5 × 100 cm). The PIs bound to the respective column were eluted with 0.1–1.0 M NaCl in 50 mM Tris-HCl, pH 8.0 (DEAE-cellulose), 0.01 N HCl (trypsin-Sepharose 4B) and 50 mM Tris-HCl, pH 8.0 (Sephadex G-50), respectively. The fractions (1.0 ml) eluted from different chromatography columns were analyzed for total protein (A_280_) and TI activity. Changes in absorbance during protein estimation and TI activity were monitored using a UV-1700 spectrophotometer (Shimadzu, Japan). The protein fractions which showed significant TI activity after each chromatography step were pooled, dialyzed and concentrated using a Labconco Freeze dryer (Cole-Parmer, USA)/Amicon filters (3 kDa cut-off) from Millipore Corporation (Billerica, USA).

### Rearing of *H. armigera* larvae

The larvae were maintained in insect culture room at 26 ± 1°C, 60 ± 5% relative humidity and 14:10 h light-dark photoperiod. Insects were fed with fresh standard artificial diet prepared according to the procedure of Gupta et al. (2000) with minor modifications. Composition of the diet for *H. armigera* was as follows (for 360 ml distilled water): 55 g chickpea seed meal, 10 g of dried yeast powder, 5 g of casein, 1.3 g of ascorbic acid, 1.0 g of methyl-*p*-hydroxybenzoate, 0.25 g of sorbic acid, 0.1 g of streptomycin sulfate, 0.055 g of cholesterol, one capsule of vitamin B-complex (~0.2 g), vitamin E (100 mg), 0.5 ml formaldehyde (40%), and 6.0 g of agar-agar.

### *In vitro* HGPs assay

The midguts from 4^th^ or 5^th^ instar larvae were dissected carefully in iso-osmotic saline (0.15 M NaCl) by narcotizing the insects on ice for 15 min. Proteases were extracted from homogenized gut tissue in two volumes of 0.05 M glycine-NaOH (pH 10.5) and centrifuged twice at 18,500 g for 10 min at 4°C (Prasad et al., 2010). The resulting supernatant was collected, aliquoted, analyzed for trypsin-like protease activity and used as a source of HGPs for *in vitro* enzyme assays and electrophoretic gelatin-activity staining studies described below. The HGPs activity was determined by monitoring the formation rate of *p*-nitroanilides from a chromogenic substrate BA*p*NA (1 mM) at 37°C in 0.05 M glycine-NaOH (pH 10.5). The molar extinction coefficient (M^−1^cm^−1^) of *p*-nitroanilide at 410 nm is equivalent to 8800.

### TI and HGPI assays

The TI activity was determined by using an appropriate volume of CpPI 63 that results in 40–60% decrease in the corresponding enzyme (trypsin) activity. Assay mixture (1.0 ml) consists of CpPI 63 in 50 mM Tris-HCl containing 20 mM CaCl_2_ at pH 8.2. Trypsin (10 μg) dissolved in HCl (0.01 N) was added to the assay mixture and incubated for 15 min at 37°C. The residual TI activity in the above assay mixture was determined after incubating for 45 min at 37°C using BA*p*NA (1.0 mM) as a synthetic substrate (Erlanger et al., 1961). Further, to monitor *H. armigera* gut trypsin-like proteinase inhibitor (HGPI) activity, an aliquot of CpPI 63, which gives 40–60% inhibition of HGPs activity in assay buffer (50 mM glycine-NaOH, pH 10.5) was incubated with *H. armigera* gut extract (an aliquot of midgut extract, which gives 1.0 optical density/45 min with 1.0 mM BA*p*NA) at 37°C for 15 min (Prasad et al., 2009). The residual HGPs activity was measured in the presence of 1 mM BA*p*NA, as a substrate. The reaction was terminated by adding 0.2 ml of 30% acetic acid (v/v). The resulting absorbance at 410 nm was recorded. One TI/HGPI unit was defined as the amount of inhibitor required to inhibit 50% of the corresponding enzyme (trypsin/HGPs) activity.

### One and 2-dimensional (2-D) gel electrophoresis

Tricine-SDS-PAGE was performed using 4% stacking gel and 18% separating gel as described by Schagger and von Jagow (1987) under non-reducing conditions (no DTT added to the protein sample).

Isoelectric focusing (IEF) was performed using Ettan IPG Phor 3 IEF system (GE Healthcare BioSciences Corp., USA) by following the manufacturer's instructions. In 2-D gel electrophoresis, IPG strips were rehydrated overnight with CpPI 63 contained in either IEF rehydration buffer (7.0 M urea, 2.0 M thiourea, 4% CHAPS and 40 mM DTT) for reducing conditions or 10% sorbitol for non-reducing conditions including 1.0% ampholytes (Swathi et al., 2014). IEF was performed at maximum current setting of 75 μA per strip by sequential steps as follows: (i) 50 V for 2 h; (ii) 500 V for 500 Vh; (iii) 1000 V for 800 Vh; (iv) 6000 V for 7000 Vh; (v) 6000 V for 2200 Vh (vi) standby mode at 500 V until removed from IEF system. After IEF, the strips were sequentially equilibrated either with DTT (50 mM) and IDA (100 mM) for 20 min each in equilibration buffer (6 M Urea, 30% Glycerol, 1 M Tris-HCl, pH 8.8, 2% SDS) followed by running buffer (50 mM Tris, 190 mM Glycine) for reducing conditions or running buffer alone for 10 min under non-reducing conditions. Second dimension was performed by tricine SDS-PAGE (18%) by using SE 600 Ruby standard dual cooled vertical unit (GE Healthcare) as described above. Proteins were detected by staining with either CBB R-250 (0.1%) or silver nitrate.

### Gelatin activity staining studies

The TI bands in native PAGE or HGPI spots in 2-D tricine SDS-PAGE were visualized by gelatin activity staining studies (Felicioli et al., 1997). Commercially available purified BBI with molecular mass 8.0 kDa was prepared (5 mg/ml) in 50 mM Tris-HCl, pH 8.0 and loaded as a reference protein. The gels performed under denaturing conditions (in the presence of SDS) were washed using 2.5% Triton-X-100 (v/v) to remove SDS. After electrophoresis, gels were incubated in 0.1 M Tris-HCl containing 0.1 mg ml^−1^ trypsin (pH 8.2) or 0.05 M Glycine-NaOH (pH 10.5) containing HGPs with activity similar to trypsin for 30 min at 4°C, followed by 2 h at 37°C. After hydrolysis of gelatin with respective proteases, the gels were washed thoroughly with distilled water to remove excess enzyme and stained with CBB R-250 (0.1%). The TI bands or HGPI spots were identified as dark blue bands/spots in a clear background due to complex formation of the unhydrolyzed gelatin with respective proteases.

### MALDI-TOF MS and ESI-MS/MS

The intact molecular mass of CpPI 63 was determined by matrix assisted laser desorption ionization time-of-flight mass spectrometry (MALDI-TOF MS) method using Autoflex III smart beam instrument (Bruker Daltonics, Bremen, Germany) equipped with a nitrogen laser (337 nm) and operated in linear mode with a matrix of α-cyano-4-hydroxy-cinnamic acid (Proteomics facility, University of Hyderabad and Sandor Lifesciences, Centre for DNA Fingerprinting and Diagnostics, Hyderabad). The spectra obtained in MALDI-TOF were analyzed using Flex analysis version 3.1 software.

The isoinhibitors resolved in 2-D PAGE were excised, reduced (50 mM DTT), alkylated (100 mM IDA) and subjected to trypsin (pI 4.8 and pI 5.1) or chymotrypsin (pI 6.6 and pI 6.1) digestion. The tryptic digested peptides were analyzed by MALDI MS/MS (Bruker Daltonics, Bremen, Germany) as described in Swathi et al. (2014). The peptides obtained from chymotrypsin digestion were analyzed by electrospray ionization mass spectrometry (ESI-MS/MS) using the Ultimate 3000 nano HPLC system (Dionex) coupled to a 4000 Q TRAP mass spectrometer (Applied Biosystems, Proteomics International Pty Ltd, Western Australia). The digested peptides were extracted according to the standard technique Bringans et al. (2008). Peptides were analyzed by ESI-MS/MS by loading onto a C18 PepMap 100, 3 μm (LC packings) and separated with a linear gradient of water/acetonitrile/0.1% formic acid (v/v). The gradient pump was programmed to deliver 5–40% of Acetonitrile over 180 min at a flow rate of 1.5 μl/min. The mobile phase “A” and “B” comprised of 0.1% formic acid in 2:98 (Acetonitrile:water), 0.1% formic acid in 95:5 (Acetonitrile:water), respectively. The obtained MS/MS spectrum for precursor ion m/z 1465.07 was analyzed by Mascot search and *de novo* sequencing was performed using PEAKS studio version 4.5 SP2 (Bioinformatics Solutions) from Proteomics International Pty Ltd, Western Australia.

### N-terminal sequencing

The N-terminal sequencing was performed by Edman's degradation method using an Applied Biosystems procise sequencer (Model No. 492) by M3 BioShodhak Pvt Ltd, Mumbai, India. The isoinhibitors (pI 6.6 and pI 5.6) of CpPI 63 are electroblotted from the 2-D gel onto a 0.22 μm PVDF membrane (sequence grade, procured from Millipore) using 10 mM CAPS buffer (pH 11.0). The elucidated peptide sequences were subjected to “BLAST” search in the NCBI database for determination of sequence homology. The closely resembled sequences were aligned using ClustalW2 software.

### Stability of TI and HGPI activity of CpPI 63 against temperature, pH and lysine modification

The effect of temperature on TI and HGPI activities of CpPI 63 was evaluated by incubating the protein at 37, 50, 75, and 90°C for 30 min. After cooling the samples to room temperature (25°C), the residual activities (TI and HGPI) were assayed at 37°C in presence of BA*p*NA as described in previous sections. The effect of pH on TI and HGPI activities of CpPI 63 was examined at pH 2.0, 8.0, and 12.0 using the following buffers at a final concentration of 50 mM: Glycine-HCl (pH 2.0), Tris-HCl (pH 8.0), and Glycine-NaOH (pH 12.0). The CpPI 63 was pre-incubated at 37°C for 1 h in the respective buffers and the residual inhibitory activities were measured against trypsin and HGPs as described above.

Lysine residues of CpPI 63 were modified using 2,4,6-trinitrobenzenesulfonic acid TNBS according to the method of Haynes et al. (1967). CpPI 63 was incubated in 50 mM phosphate buffer (pH 7.6) with 5, 10, 15, and 20-fold molar excess of TNBS at 40°C for 2 h. The reaction was stopped by the addition of 10% SDS. The residual TI and HGPI activities were evaluated using BA*p*NA as substrate as described in previous sections.

### Statistical analysis

The experiments conducted in the present study were carried out at least three or four times each with three replications and the mean ± SE was reported. Statistical differences were determined by one-way ANOVA followed by Tukey test at *P* ≤ 0.05 using Sigma-Plot, version 11.0, software (San Jose, CA, USA).

## Results

### Purification of CpPI 63

**Figure 1:**
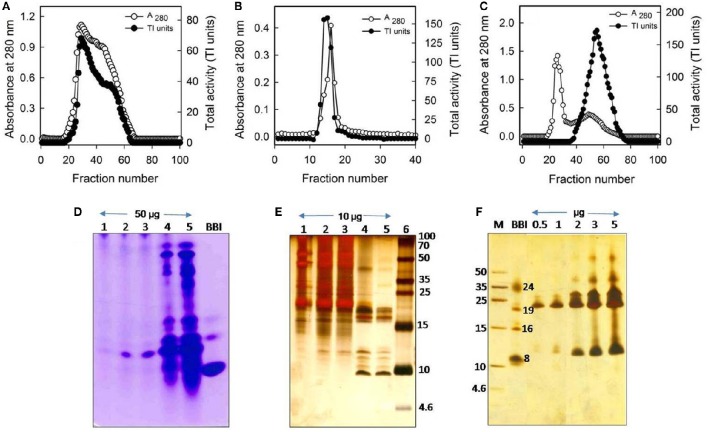
**Purification profile of trypsin-specific PIs (CpPI 63), Gelatin native PAGE, and Tricine SDS-PAGE**. Elution profile of **(A)** DEAE-cellulose column loaded with 20–70% (NH_4_)_2_SO_4_ fraction; **(B)** trypsin-Sepharose 4B column loaded with PI fraction (No. 20–63) pool from the ion-exchange column; **(C)** Sephadex G-50 column loaded with PI fraction (No. 12–18) pool from the affinity column; **(D)** activity staining of Gelatin native PAGE (12.5%) and; **(E)** Tricine SDS-PAGE (18%) showing different fractions of the purification protocol. Lanes 1-6 are loaded with crude PI protein extract, 20–70% (NH_4_)_2_SO_4_ protein fraction, active fraction pool against trypsin from anion exchange column, affinity column, gel-filtration column (fraction No. 38–73), Marker protein, soybean BBI (2 μg) or Puregene molecular weight marker, respectively. Gels were stained with CBB R250 stain or silver nitrate; **(F)** Tricine SDS-PAGE (18%) showing the consequent increase in intensity of PI bands and appearance of their oligomers with the progressive increase in concentration of CpPI 63 (lanes 3–7). Lanes 1 and 2 are loaded with molecular weight marker (Puregene) and soybean BBI (2 μg), a PI marker, as the mobility of PIs is known to vary as compared to other proteins. The molecular mass of monomeric (8 kDa), dimeric (16 kDa), and trimeric (24 kDa) forms of BBI is indicated. An additional band at ~19 kDa might be a contamination of Kunitz inhibitor in BBI preparation, which is procured commercially. The data shown here are representative of a purification protocol for CpPI 63 from four experiments performed independently as described in Table 1.

The PIs from *C. platycarpus* with trypsin-specific activity were purified by passing the (NH_4_)_2_SO_4_ precipitated protein sequentially through three different chromatography columns. It was found that 20–70% (w/v) (NH_4_)_2_SO_4_ saturated protein fraction possessed significant inhibitory activity against trypsin when compared with other (NH_4_)_2_SO_4_ saturated fractions (0–20 and 70–100%). The elution profile of the 20–70% (NH_4_)_2_SO_4_ fraction from the DEAE-Cellulose chromatography is represented in Figure 1A. The eluted fractions (#20–63) containing TI activity were further purified on a trypsin-Sepharose affinity chromatography (Figure 1B). The protein fractions (#12–18) from a sharp peak of the affinity chromatography with significant TI activity were passed through Sephadex G-50 gel filtration chromatography to remove high molecular weight contaminants (Figure 1C). Finally, the eluted protein fractions (#38–73) from gel filtration chromatography, which were enriched in trypsin-specific PIs were pooled and labeled as “CpPI 63,” and stored at −20°C until further use. The CpPI 63 which is purified by using the above protocol resulted in < 22-fold purification and < 7% yield recovery (Table 1). The TI activity of purified CpPI 63 was ascertained by activity staining studies (Figure 1D). The progressive increase in the color intensity of the bands on gelatin Native PAGE clearly represent the enhanced purity of the CpPI 63 during sequential steps of purification applied in the present study. Further, the SDS-PAGE analysis of CpPI 63 under non-reducing conditions showed several bands with an apparent molecular mass range of 10–56 kDa (Figures 1E,F). An increase in number of isoinhibitors and self-association pattern was observed in tricine SDS-PAGE with an increase in loading concentration of CpPI 63 from 0.5 to 5.0 μg (Figure 1F). Bowman-Birk inhibitor (BBI) was loaded parallel to molecular weight markers as the electrophoretic mobility of PIs is known to vary as compared to molecular weight markers (Swathi et al., 2014).

**Table 1:** **Purification of trypsin-specific PIs “CpPI 63” from mature seeds of *C. platycarpus***.

**Purification step**	**Total protein (mg)**	Total activity (^*^TI units)	**Yield recovery (%)**	**Specific activity (TI units/mg protein)**	**Purification (Fold)**
Crude PI protein extract	254	4139	100	16.3	1.0
(NH4)2SO4 fraction (20–70%)	57	3245	78.4	56.9	3.5
Ion-exchange chromatography	50	1478	35.6	29.5	1.8
Affinity chromatography	4.0	738	17.8	185	11.4
Gel-filtration chromatography	0.8	286	6.9	358	21.7

*The results are representative of four independent experiments performed with seed material collected from two different batches grown in greenhouse*.

* *One TI unit is defined as the amount of CpPI 63 required to inhibit 50% of BApNA hydrolysis by trypsin*.

### CpPI 63 exist as small oligomers and isoinhibitors

**Figure 2:**
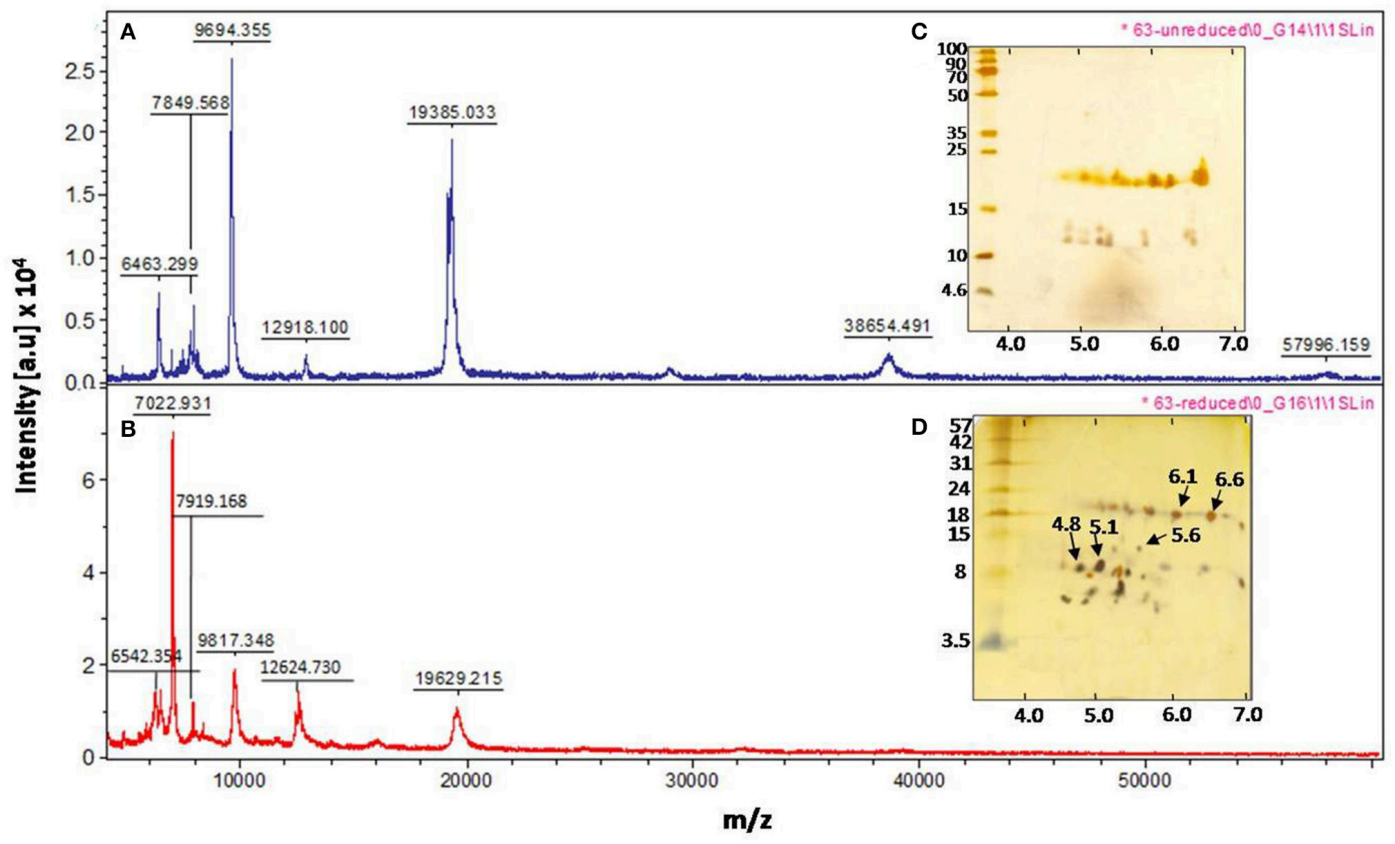
**Intact mass analysis of different isoinhibitors of CpPI 63 purified from mature seeds of *C. platycarpus* using MALDI-TOF mass spectrometer and 2-D electrophoresis**. MALDI-TOF mass spectrum of CpPI 63 between m/z 0–60000 under non-reducing **(A)** and reducing conditions **(B)** i.e., after reduction and alkylation with DTT (50 mM) and IDA (100 mM), respectively; Separation of isoinhibitors of CpPI 63 (25 μg) in 2-D electrophoresis under non-reducing **(C)** and reducing conditions **(D)**. Iso electric focusing (IEF) was performed using pH 4–7, 11 cm strips as described in materials and methods. Second dimension was executed using tricine SDS-PAGE (18%). The data shown here are representative of three experiments performed independently with CpPI 63 purified from different experiments.

Correlating with the self-association (oligomeric) nature of CpPI 63 in SDS-PAGE and detection of several trypsin-specific bands in gelatin native PAGE, the intact mass analysis of CpPI 63 also revealed several peaks as dimer, trimer, and tetramer in MALDI-TOF under non-reducing conditions where the PI was not reduced with DTT (Figures 1D–F, 2A). In general, the self-association nature of proteins with trypsin inhibitory activity is associated with either miraculin-like proteins (MLPs) or BBIs. Further, the presence of multiple PIs in CpPI 63, as observed in MALDI-TOF and activity staining studies was also detected in 2-D electrophoresis under both native and reducing conditions (Figures 1D–F, 2A–D). Several isoinhibitors with a molecular mass of ~19 kDa were resolved along with low molecular weight proteins between pH 4.0 and 7.0 in 2-D PAGE under native and reducing conditions (Figures 2C,D). Further, the difference in mass (244.182 Da) of a peak at m/z 19385.033 under non-reducing conditions to a peak at m/z 19629.215 after reduction and alkylation with DTT and iodacetamide (57 Da), respectively, suggests the presence of a TI with two disulphide bonds, a characteristic feature of Kunitz trypsin inhibitors (KTIs) as shown in Figures 2A,B (Prasad et al., 2010).

### Identification of isoinhibitor spots PI 6.6 and PI 5.6

The MALDI MS/MS analysis was performed on the trypsin digested isoinhibitor spots with pI 4.8 and pI 5.1(data not shown), while ESI MS/MS analysis was performed on the chymotrypsin digested isoinhibitor spots with pI 6.6 and pI 6.1 (data not shown), obtained from 2-D gel under reducing conditions (Figure 2D). However, the MS/MS spectrum of the precursor ion m/z 1465.07 obtained from the isoinhibitor spot pI 6.6 is shown in Figure S1A. As the above spectrum did not show any sequence homology with the reported TIs in database search (Figure S1B), *de novo* sequencing was performed. This could be possible as the genome sequences of *C. platycarpus* were not available so far in the public databases. The fragment ions and relevant monoisotopic masses of the generated “*b”* and “*y”* ions for the *de novo* sequence are shown in Figure S1C. The *de novo* sequence “LLLVAPPPGPAVAHL” generated from the precursor ion m/z 1465.07 showed partial homology with a few PIs in the following order: Xylanase inhibitor > Amylase inhibitor > Kunitz trypsin inhibitor > Squash aspartic acid proteinase inhibitor (Figures S1D,E).

**Figure 3:**
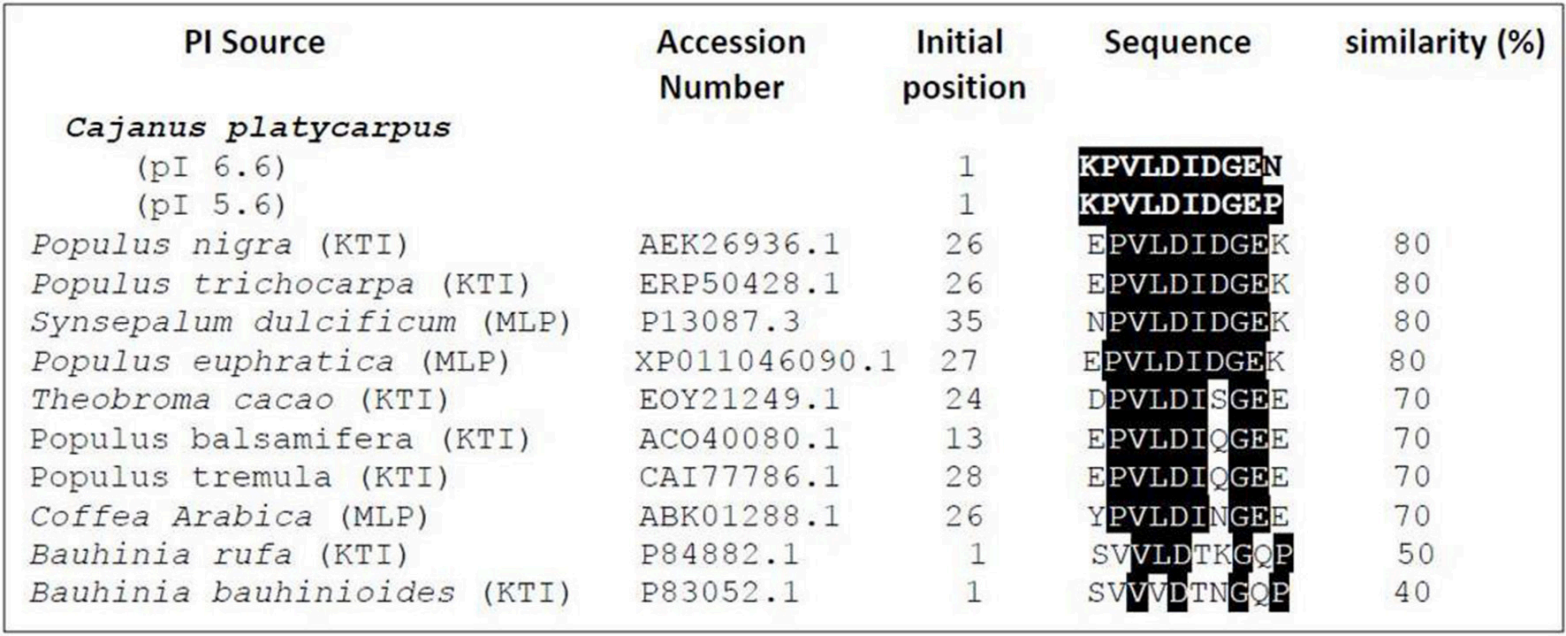
**Alignment of N-terminal sequences of isoinhibitor spots of CpPI 63**. The isoinhibitor spots with pI 6.6 (KPVLDIDGEN) and pI 5.6 (KPVLDIDGEP) separated in 2-D gel under reducing conditions showed homology with other known Kunitz-type trypsin inhibitors from *P. nigra* (Nr: AEK26936.1); *P. trichocarpa* (Nr: ERP50428.1); *T. cacao* (Nr: EOY21249.1); *P. balsamifera* (Nr: ERP50428.1*); P. tremula* (Nr: CAI77773.1); *B. rufa* (Nr: P84882.1); *B. bauhinioides* (Nr: P83052.1) as well as miraculin-like proteins from *S. dulcificum* (Nr: P13087.3); *C. arabica* (Nr: ABK01288.1); *P. euphratica* (Nr: XP_011046090.1). The search criterion was limited with “Non-redundant protein sequences” and Plants (Taxid: 3193).

The N-terminal sequencing of isoinhibitor spots (pI 6.6 and pI 5.6) was carried out using Edman's degradation method after eluting from the 2-D gel performed under reducing conditions (Figures 2D, 3). The N-terminal sequences “KPVLDIDGEN” and “KPVLDIDGEP” of the isoinhibitor spots pI 6.6 and pI 5.6, respectively, showed similarity with a conserved signature pattern of the PIs belonging to the KTIs family as well as MLPs. The primary amino acid sequence of MLPs possessed a conserved motif [LIVM]-x-D-x-[EDNTY]-[DG]-[RKHDENQ]- x-[LIVM]-(x)5-Y-x-[LIVM] which is similar to the soybean KTIs family (McLachlan, 1979; Srinivasan et al., 2005). Thus, the above N-terminal sequences showed 80% homology with both reported KTIs and MLPs as well (Figure 3).

### Inhibitory activity of CpPI 63 against HGPs

#### Specific activity and IC_50_

**Figure 4:**
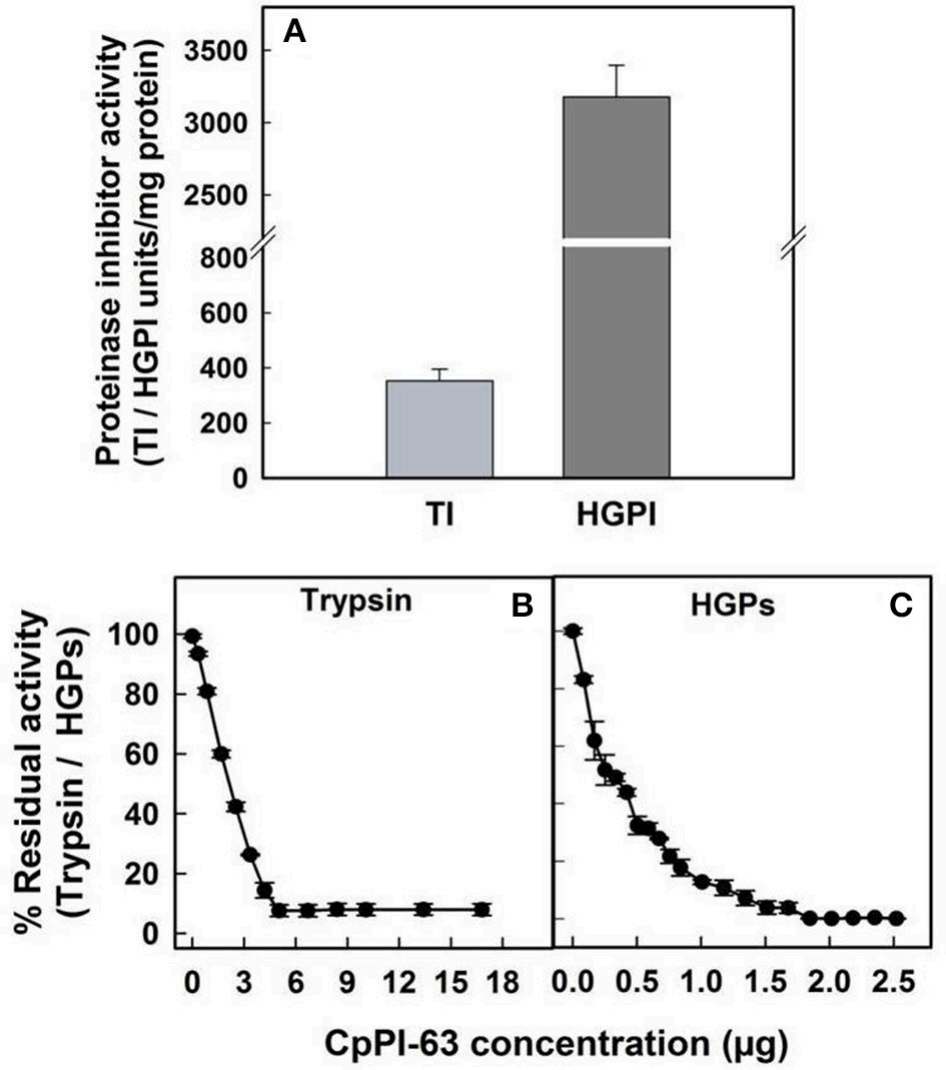
**Evaluation of inhibitory activity of CpPI 63 against HGPs**. The specific activities of CpPI 63 against bovine pancreatic trypsin and HGPs **(A)**; half maximal inhibitory concentrations (IC_50_) of CpPI 63 against **(B)** bovine pancreatic trypsin and **(C)** HGPs. The experiments were conducted three times, each with three replications and the mean ± S.E was reported.

In lepidopteran insects such as *H. armigera*, serine proteases are the major group of hydrolytic enzymes involved in the physiological process of digestion (Srinivasan et al., 2006). Among the serine proteases, the activity of trypsin-like proteases of *H. armigera* (HGPs) is several folds higher over chymotrypsin-like proteases (Swathi et al., 2015). Therefore, in the present study, the inhibitory potential of CpPI 63 was examined toward HGPs alone. The specific activity of CpPI 63 against HGPs (3178 HGPI units/mg protein) was 9-fold higher than the specific activity against bovine pancreatic trypsin (353 TI units/mg protein) which is used as a reference standard (Figure 4A). Also, as indicated in Figures 4B,C, the CpPI 63 exhibited strong inhibitory potential against HGPs (IC_50_ of 240 ng) as compared to bovine pancreatic trypsin (IC_50_ of 2.1 μg). Thus, the amount of CpPI 63 used to attain the maximum (100%) inhibition of HGPs (1.8 μg) is much lower than the bovine pancreatic trypsin (5.4 μg).

#### Gelatin activity staining

**Figure 5:**
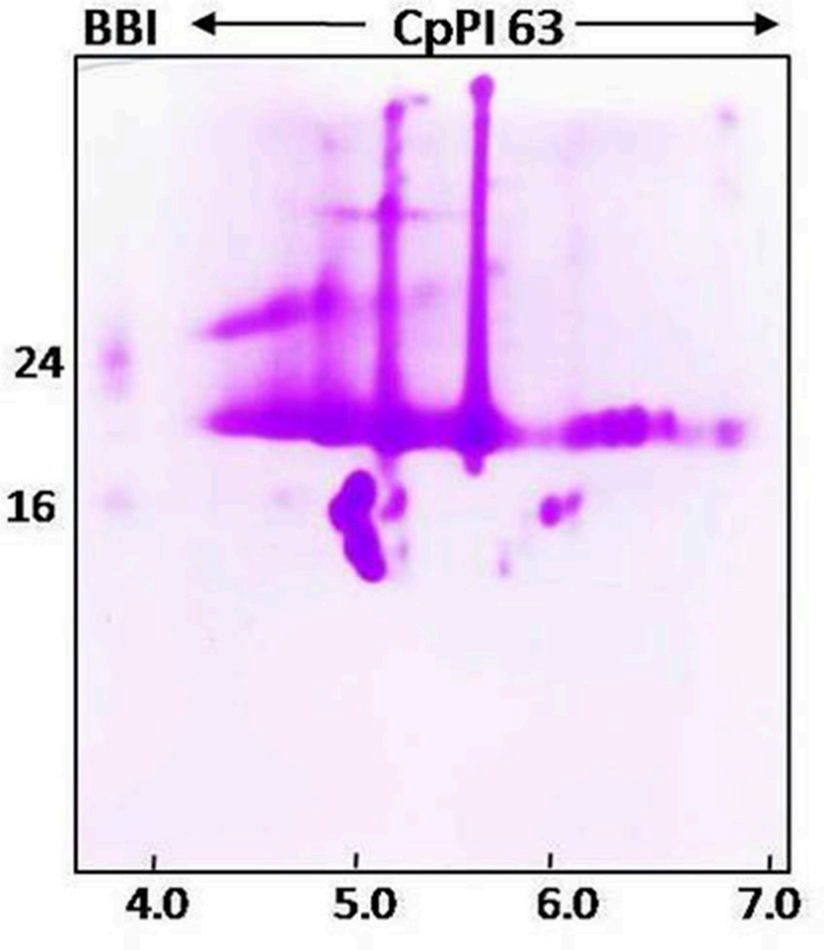
**Activity staining of different isoinhibitor spots of CpPI 63 against HGPs**. CpPI 63 (75 μg) was subjected to Iso electric focusing (IEF) under non-reducing conditions as described in materials and methods using pH 4–7, 11 cm strips. Second dimension of electrophoresis was executed using gelatin SDS-PAGE (18%). After electrophoresis, the gel was incubated with Triton-x-100 followed by HGPs as described in materials and methods and stained with CBB R250 to visualize the isoinhibitor spots of CpPI 63 active against HGPs. The gel shown here is a selective representative from three replicates of CpPI 63 purified from three independent experiments.

Since CpPI 63 was resolved into several inhibitor spots with specific molecular mass and iso-electric points in 2-D electrophoresis, gelatin activity staining was performed under native conditions to ensure the inhibitory activity of these resolved isoinhibitors and/or oligomeric forms against HGPs (Figures 1D,F, 2C,D, 5). However, in native conditions (without DTT reduction of CpPI 63) all the inhibitor spots and its oligomers exhibited strong inhibitory activity against HGPs (Figure 5). In general, the MLPs as well as BBIs exist in oligomers such as homo dimers, trimers and tetramers. However, the appearance of multiple PIs with a unique pattern of expression in CpPI 63 of *C. platycarpus* accession and their significant inhibitory potential against HGPs might be responsible to counteract the diversified midgut proteases of polyphagous insect pests like *H. armigera* (Figures 1D, 2A–D, 5).

#### Stability studies

**Figure 6:**
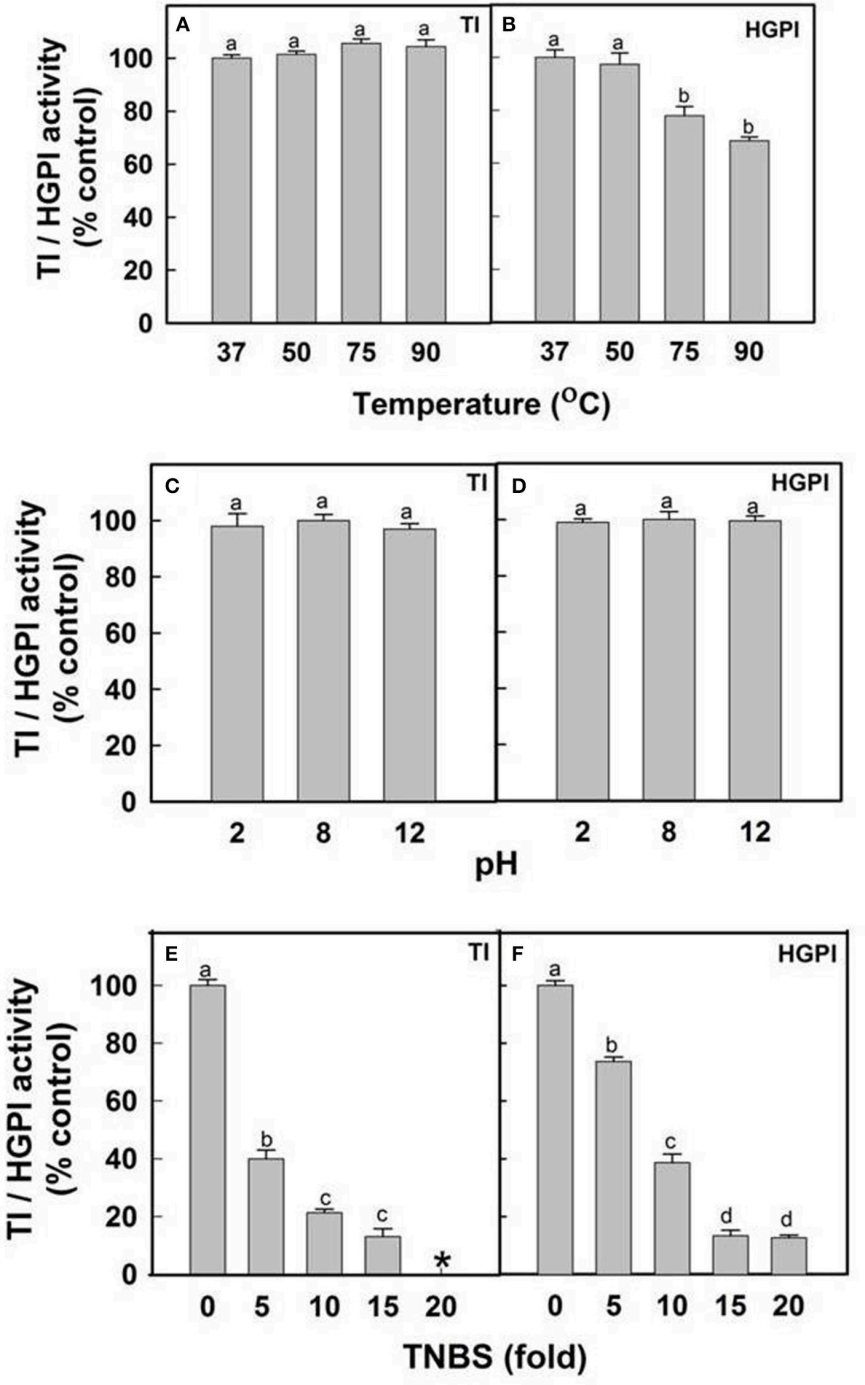
**Effect of temperature, pH and lysine modification on HGPI activity of CpPI 63**. Stability of TI/HGPI activities of CpPI 63 against **(A)** bovine pancreatic trypsin and **(B)** HGPs, respectively, after incubating for 30 min at indicated temperatures before performing the respective assays; Stability of TI/HGPI activities of CpPI 63 against **(C)** bovine pancreatic trypsin and **(D)** HGPs, respectively, after incubating with respective pH buffers at 37°C for 1 h before performing the assays; Loss in the TI/HGPI activities of CpPI 63 against **(E)** bovine pancreatic trypsin and **(F)** HGPs, respectively, after biochemical modification of lysine residues in it using TNBS as described in materials and methods. ^*^indicate complete loss of inhibitory activity against trypsin. Statistics were performed as described in Figure 4. Different lowercase alphabetical letters indicate statistically significant differences (*P* < 0.05).

The stability of inhibitory activity of CpPI 63 against bovine pancreatic trypsin and HGPs was examined in a wide range of temperature and pH conditions (Figures 6A–D). The TI and HGPI activities of CpPI 63 showed 100% stability when incubated up to 90 and 50°C, respectively. However, the HGPI activity was decreased by ~23% at 75°C and ~32% at 90°C (Figures 6A,B). In contrast, both the TI and HGPI activities of CpPI 63 were stable at both acidic (2.0) and basic (8.0 and 12.0) pH with a marginal loss (5%) in activity (Figures 6C,D).

Further, in chemical modification studies, a chemical reagent is used to bind covalently to specific amino acid side chains of a protein which in turn produce changes in the activity of a protein. Attempts to correlate these changes with catalytic activity have been made previously (Prasad et al., 2010; Swathi et al., 2014). In the present study lysine residues of CpPI 63 were modified using a chemical reagent 2,4,6-trinitrobenzene sulfonic acid (TNBS). It is a sensitive reagent which specifically modifies the free ε amino group of L-lysine residue to highly chromogenic derivative. As a result, the TI and HGPI activities of CpPI 63 were gradually decreased by increasing the concentration of TNBS by 5–20-fold as compared to the concentration of CpPI 63 (Figures 6E,F). A complete loss in TI and 80% loss in HGPI activity was observed when the TNBS concentration was raised to 20-fold (Figures 6E,F). These results indicate the importance of lysine residues at the reactive site of the CpPI 63.

## Discussion

The protein digestion in lepidopteran larvae mainly relies on serine proteases such as trypsin and chymotrypsin (Srinivasan et al., 2006). Therefore, the use of PIs targeting digestive proteases of insect pests is one of the beneficial approaches used in the integrated pest management. The lepidopteran pod borer *H. armigera* is a major biotic constraint on the production of pigeonpea. Several earlier reports indicated that the PIs prepared from crude protein extracts of wild relatives of pigeonpea significantly inhibited the activity of HGPs (Chougule et al., 2003; Parde et al., 2012; Swathi et al., 2015). Therefore, in the present study, trypsin-specific PIs (CpPI 63) active against HGPs were purified from mature dry seeds of *C. platycarpus* using different chromatography methods and characterized to complement the earlier findings.

The appearance of several TI bands (polymorphic profile) in gelatin Native PAGE of CpPI 63 is in agreement with other wild relatives of pigeonpea and crop plants such as *C. volubilis, C. crassicaulis*, and *C. scarabaeoides* (Kollipara et al., 1994; Chougule et al., 2003; Prasad et al., 2009); *Cicer arietinum, Glycine max*, and *Vigna ceratotropis* (Patankar et al., 1999; Konarev et al., 2002a,b; Krishnamurthy et al., 2013), respectively. In contrast, the electrophoretic appearance of PIs in cultivars is highly homogenous possibly due to the loss of such genetic diversity during the course of domestication (Chougule et al., 2003; Prasad et al., 2009, 2010; Swathi et al., 2014). Thus, the existence of several TIs with HGPI activity could be responsible for the widespread resistance against *H. armigera* in *C. platycarpus* or other wild relatives/crop plants (Pichare and Kachole, 1996; Saxena et al., 1996; Sujana et al., 2008).

The molecular mass range of various TIs in CpPI 63 observed in SDS-PAGE (10–56 kDa) is apparently in accordance with the MALDI TOF mass spectrum (6–58 kDa) under native conditions. The appearance of a major peak at m/z 19385.033 under native conditions and an increase in its mass by 244.18 Da on treatment with DTT and IDA indicate the presence of TIs with two disulphide bonds in CpPI 63, a characteristic feature of KTIs (Prasad et al., 2010; Macedo et al., 2015). Further, the TIs of CpPI63 were resolved as several isoinhibitors in the acidic pH range. Similar results were reported in *Psophocarpus tetragonolobus* (WbTI) where 14 isoinhibitors with mass variation of 6–28 kDa are resolved in 2-D PAGE. Four of these isoinhitors resembled KTIs while three showed similarity with BBIs (Giri et al., 2003). The occurrence of several isoinhibitors might have evolved from gene duplication, multi-gene products or post translational modifications during co-evolution of plants and insects to counteract each other (Zhu-Salzman and Zeng, 2015). Interestingly, in the present study, all the isoinhibitors (HGPIs) and their respective oligomers resolved in 2-D activity PAGE exhibited strong inhibitory activity against HGPs. Also, the observed oligomeric pattern of HGPIs in 2-D electrophoresis corroborated well with the self-association pattern of TIs resolved in tricine SDS-PAGE. Thus, the different PIs, which exist within the same plant species might vary in their number, size, family, and specificity toward the protease(s) with which they interact (Dunse et al., 2010; Jamal et al., 2013; Botelho-Junior et al., 2014).

In 2-D PAGE under reducing conditions, several new low molecular weight isoinhibitor spots appeared in concurrence with the disappearance of several high intensity isoinhibitor spots at ~19 kDa region under native conditions. These results imply that isoinhibitors of CpPI 63 present at ~19 kDa region might exist in a combination of both as a single as well as two polypeptides chain isoinhibitors (Ee et al., 2009; Rufino et al., 2013). The identification of high molecular mass TIs in SDS-PAGE, MALDI intact mass analysis, and 2-D activity staining studies together suggest the possibility of existence of MLPs in CpPI 63. Similar to KTIs, MLPs also belonged to Kunitz super family and exhibited inhibitory activity against trypsin and HGPs (Gahloth et al., 2011). In contrast to KTIs, MLPs exist as oligomers by inter molecular disulphide bond, which is evident by the disappearance of high molecular mass TIs in CpPI 63 on reduction with DTT in MALDI-TOF studies and 2-D electrophoresis.

N-terminal analysis of CpPI 63 was performed in order to eliminate ambiguity in protein identity of the *de novo* sequence “LLLVAPPPGPAVAHL” obtained from ESI MS/MS analysis. The N-terminal sequences of CpPI 63 (pI 6.6 and pI 5.6) exhibited 80% homology with the conserved sequence of both KTIs and MLPs. Norioka et al. (1988) screened for PIs in various leguminous plants and reported that the seeds of very primitive species such as wild relatives contained primarily KTIs, and during the course of evolution, KTIs have been systematically remolded into BBIs such as in pigeonpea cultivars ICP 14770 (Prasad et al., 2010), ICP 7118 (Swathi et al., 2014) and TAT-10 (Godbole et al., 1994). Apart from KTIs, the PIs, which belong to BBIs, Squash, α-Amylase/trypsin, Aspartic or Mustard trypsin inhibitor families also exhibit TI activity (Macedo et al., 2015).

The retention of HGPI activity (~70%) by CpPI 63 at both high temperature (90°C) and a wide range of pH suggests its structural and functional stability, particularly against high alkaline pH that exist in the insect gut environment (Shaikh et al., 2014). In general, the endurance of PIs to high temperature is associated with several intra and inter molecular disulphide bonds as well as electrostatic and van der Waal's interactions generated due to the differential distribution/composition of both hydrophobic and hydrophilic amino acids (Kumar et al., 2004). The studies of Hogg (2003) reported that disulphide bonds have been added to the PIs during evolution to enhance the protein stability, which in turn prevent their digestion by cellular proteases and thereby increase their half-life by molecular packing. Stability against high temperature and pH was also observed with other group of PIs from seeds of *Dolichus biflorus* (Kumar and Gowda, 2013), *Pithecellobium dumosum* (Rufino et al., 2013), and *Putranjiva roxburghii* (Chaudhary et al., 2008).

Lysine is one of the important amino acid present in the reactive site as P_1_ in KTIs and BBIs (Chauvet and Acher, 1975; Lin et al., 1991; Oliva et al., 1996; Kumar et al., 2004; Liao et al., 2007; Prasad et al., 2010). Modification of Lysine residues resulted in gradual loss in the inhibitory activity of CpPI 63 against trypsin and HGPs, possibly due to a conformational change in P_1_ residue (direct effect) or a disturbance in the interaction with the target enzyme generated due to a loss in their 3-D structure (indirect effect). Thus, considering the presence of several isoinhibitors with significant (i) HGPI activity; (ii) IC_50_ (480 ng) against HGPs and; (iii) stability toward proteolytic degradation by HGPs at high temperature and alkaline pH, CpPI 63 can be exploited in the management of *H. armigera*.

The results from the present study suggest that wild relative of pigeonpea, *C. platycarpus* (ICPW 63) is a rich source of trypsin-specific PIs “CpPI 63” which possesses strong inhibitory activity against HGPs. The MALDI TOF and 2D-electrophoresis of CpPI 63 demonstrated the existence of trypsin-specific PIs as several isoinhibitors and small oligomers. The N-terminal sequencing of CpPI 63 isoinhibitors separated at pI 6.6 and pI 5.6 in 2-D gel suggest that they belong to either KTIs and/or MLPs. Further studies are warranted to understand the protein identity of the various other isoinhibitor spots of CpPI 63 separated by 2-D gel so as to develop strategies for producing pigeonpea cultivars resistant against *H. armigera*.

## Author contributions

Conceived and designed the experiments: KP; Experimental material: NM; Performance of the experiments: MS, PM, VL and VS; Analysis of the data: MS and KP; Manuscript writing: MS; Manuscript editing: KP; Critical suggestions during experimental design and manuscript editing: AD.

### Conflict of interest statement

The authors declare that the research was conducted in the absence of any commercial or financial relationships that could be construed as a potential conflict of interest.

## Supplementary material

Figure S1 **The ESI MS-MS analysis of isoinhibitor spot (pI 6.6) of CpPI 63 separated in 2-D gel under reducing conditions. (A)** The representative fragmentation spectrum of precursor ion m/z 1465.07 generated after digestion of isoinhibitor spot with chymotrypsin; **(B)** The details of Mascot search corresponding to ESI-MS/MS of precursor ion m/z 1465.07; **(C)** Fragmentation table showing the “b” and “y” ion series matching the spectrum along with the mass differences of the ions; **(D)** the corresponding *de novo* sequence “LLLVAPPPGPAVAHL” and **(E)** The alignment of *de novo* amino acid sequence with related proteins: Xylanase inhibitor from Japanese rice *Oryza sativa* (Nr:Q53NL5.1; GI:73622089); Amylase inhibitor from *Zea mays* (Nr:1BEA_A; GI:157830250); Kunitz trypsin inhibitor from *Glycine max* (Nr:1BA7_A; GI:3318877); Squash aspartic acid proteinse inhibitor from *Cucurbita maxima* (Nr:2KXG_A; GI:297787502). The white letters on the black background indicate the identical amino acid residues. The search criterion was limited with “Non-redundant protein sequences” and Plants (Taxid: 3193). Also, it is possible to have isoleucine in place of leucine in the *de novo* sequence due to similarity in their mass. However, no homology was detected when databases were searched by replacing isoleucine with leucine.

## Abbreviations

BBI: Bowman-Birk inhibitor
*C. platycarpus*: *Cajanus platycarpus*
*C. cajan*: *Cajanus cajan*
*H. armigera*: *Helicoverpa armigera*
HGPs: *H. armigera* gut trypsin-like proteases
HGPIs: *H. armigera* gut trypsin-like proteinase inhibitors
KTIs: Kunitz trypsin inhibitors
MLPs: Miraculin-like proteins
PIs: Proteinase inhibitors
TIs: Trypsin inhibitors.
