# Supplementary figures and images for: Purification and Partial Characterization of Trypsin-Specific Proteinase Inhibitors from Pigeonpea Wild Relative Cajanus platycarpus L. (Fabaceae) Active against Gut Proteases of Lepidopteran Pest Helicoverpa armigera
Source: Front Physiol. 2016 Sep 7;7:388. doi: 10.3389/fphys.2016.00388 (PMC5013044; doi:10.3389/fphys.2016.00388)

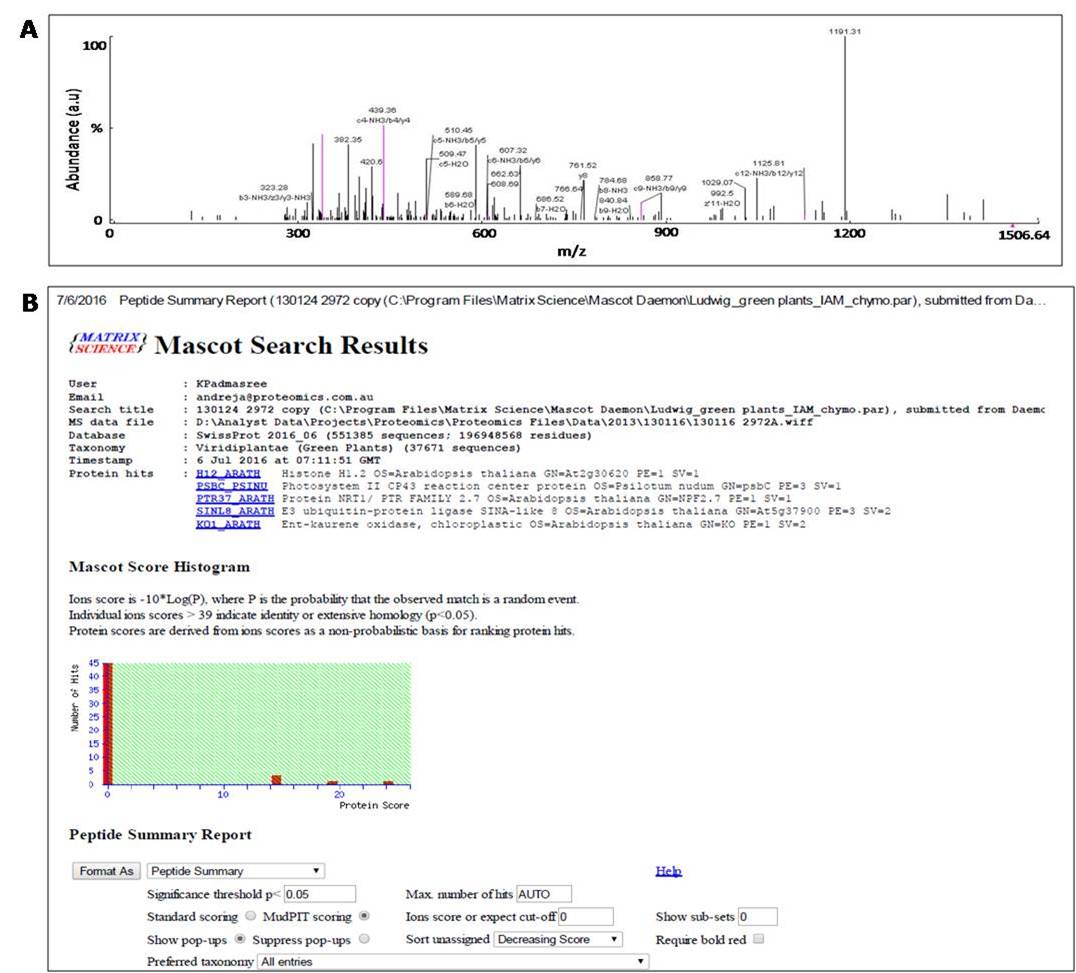

Supplement: Figure S1 — The ESI MS-MS analysis of isoinhibitor spot (pI 6.6) of CpPI 63 separated in 2-D gel under reducing conditions. (A) The representative fragmentation spectrum of precursor ion m/z 1465.07 generated after digestion of isoinhibitor spot with chymotrypsin; (B) The details of Mascot search corresponding to ESI-MS/MS of precursor ion m/z 1465.07; (C) Fragmentation table showing the “b” and “y” ion series matching the spectrum along with the mass differences of the ions; (D) the corresponding de novo sequence “LLLVAPPPGPAVAHL” and (E) The alignment of de novo amino acid sequence with related proteins: Xylanase inhibitor from Japanese rice Oryza sativa (Nr:Q53NL5.1; GI:73622089); Amylase inhibitor from Zea mays (Nr:1BEA_A; GI:157830250); Kunitz trypsin inhibitor from Glycine max (Nr:1BA7_A; GI:3318877); Squash aspartic acid proteinse inhibitor from Cucurbita maxima (Nr:2KXG_A; GI:297787502). The white letters on the black background indicate the identical amino acid residues. The search criterion was limited with “Non-redundant protein sequences” and Plants (Taxid: 3193). Also, it is possible to have isoleucine in place of leucine in the de novo sequence due to similarity in their mass. However, no homology was detected when databases were searched by replacing isoleucine with leucine. [file Image1.JPEG]

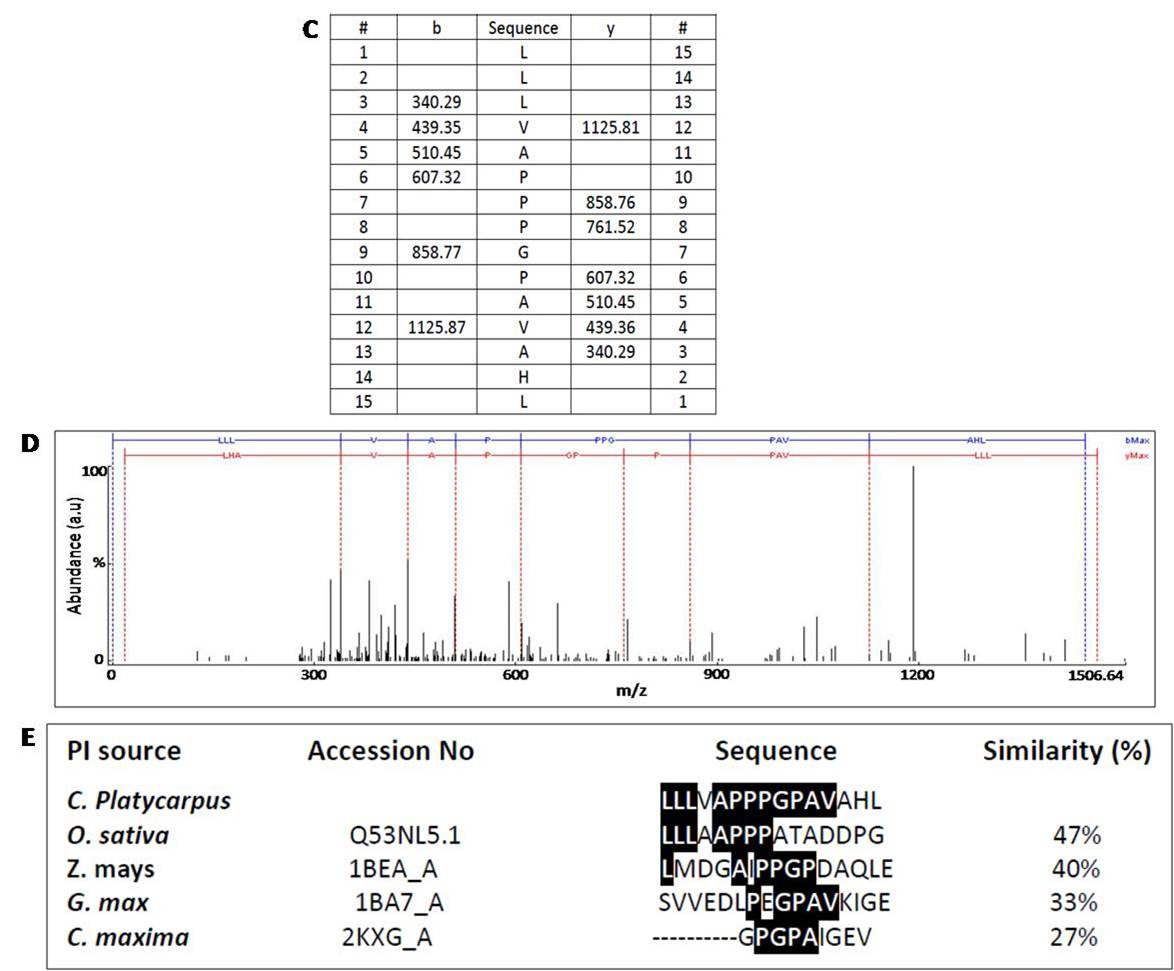

Supplement: Supplementary file 2 [file Image2.JPEG]
